# Extent and Predictors of Poor Glycaemic Control among Elderly Pakistani Patients with Type 2 Diabetes Mellitus: A Multi-Centre Cross-Sectional Study

**DOI:** 10.3390/medicina55010021

**Published:** 2019-01-17

**Authors:** Muhammad Atif, Quratulain Saleem, Saima Asghar, Iram Malik, Nafees Ahmad

**Affiliations:** 1 Department of Pharmacy, The Islamia University of Bahawalpur, Bahawalpur 63100, Pakistan; q.pharma@gmail.com (Q.S.); saimaasghar93@yahoo.com (S.A.); iramalik686@gmail.com (I.M.); 2 Faculty of Pharmacy and Health Sciences, University of Balochistan, Quetta 87300, Pakistan; nafeesuob@gmail.com

**Keywords:** diabetes, type 2 diabetes mellitus, elderly, glycaemic control, HbA1c levels, Pakistan

## Abstract

*Objectives:* This study aimed to explore the relationship between glycaemic control and factors that may influence this among elderly type 2 diabetes mellitus (T2DM) patients in Lahore, Pakistan. *Methods:* This descriptive, cross-sectional study was conducted at the Jinnah and Sir Ganga Ram Hospitals, Lahore using convenience sampling techniques between 1 December 2015 and 28 February 2016. The sample consisted of elderly (>65 years) T2DM patients. Glycaemic values and patient characteristics were obtained from medical charts. Consenting patients were interviewed to complete the Barthel Index, Lawton Instrumental Activities of Daily Living Scale, Clinical Frailty Scale, Iowa Pain Thermometer Scale, Geriatric Depression Scale, Montreal Cognitive Assessment tool, Mini Nutritional Assessment Scale—Short Form and Self Care Inventory—Revised Version. Multiple logistic regression analysis was carried out to determine the predictors of poor glycaemic control. *Results:* A total of 490 patients were approached and 400 agreed to participate. Overall, nearly one-third (32.2%, *n* = 129) of patients had glycated haemoglobin (HbA1c) at the target level. Fasting and random plasma glucose levels were within the target range to much the same extent; (36.8%, *n* = 147) and (27%, *n* = 108), respectively. HbA1c levels were also higher in patients with co-morbidities (67.4%, *n* = 229) with diabetes-related complications (73.5%, *n* = 227). Significant predictors of impaired glycaemic control (HbA1c) included poor diabetes self-care (adjusted odds ratio (AOR) 0.96; 95% confidence interval (CI) 0.95, 0.98), not being prescribed oral hypoglycaemic agents (OHA) (AOR 6.22; 95% CI 2.09, 18.46), regular hypoglycaemic attacks (AOR 2.53; 95% CI 1.34, 4.81) and falling tendency (AOR 0.19; 95% CI 0.10, 0.36). *Conclusions:* Poor glycaemic control prevailed among the majority of elderly Pakistani diabetic patients in this study. Triggering factors of poor glycaemic control should be taken into consideration by the healthcare professionals in targeting multifaceted interventions to achieve good glycaemic control.

## 1. Introduction

Diabetes in the elderly imposes significant individual and community level health burdens [1]. Type 2 diabetes mellitus (T2DM) is rapidly rising among older adult populations due to the compounding effects of developing insulin resistance and impaired functioning of pancreatic islet cells with increasing age [2]. In 2017, global estimates report that diabetes affects almost 425 million people worldwide (20−79 years of age), of which close to one-third (122.8 million) are older than 65 years. If the diabetes trend continues on the same trajectory, it will affect an estimated 629 million people (20−79 years of age) by 2045, of which 253.4 million will be elderly patients (65–99 years of age). Approximately, 3.2 million elderly patients (60–99 years of age) die from diabetes worldwide each year (60% of all deaths due to diabetes among the 18–99 age group) [3].

The elderly have distinctive health needs and their own biomedical, mental, and societal constitution [4]. The widespread prevalence of diabetes in the elderly population is characterized by the complexity of illness and emergent co-morbidities [5]. At the same time, elderly diabetic patients are at increased risk of developing macro-vascular (cardiovascular disorders) and micro-vascular complications (e.g., retinopathy, neuropathy, nephropathy) [3,5]. Glycaemic control is vital to diabetes management, which necessitates frequent screening of blood glucose levels, ophthalmic follow up, renal function tests, standard diet plans, podiatry foot care, exercise and modified pharmaco-therapy [6,7]. Glycaemic control in T2DM patients can be evaluated through three key parameters: glycated haemoglobin (HbA1c), fasting plasma glucose (FPG) and random plasma glucose (RPG). However, HbA1c (level < 7%) is the gold standard for evaluating glycaemic control [8,9]. Impaired glycaemic control can significantly increase healthcare costs and minimize quality and expectancy of life [10,11]. According to one estimate, a 1% decline in HbA1c level results in a 21% and 37% decreased risk of diabetes-associated mortality and micro-vascular complications, respectively [11]. 

Studies report that significant numbers of diabetic patients have poor glycaemic control and older age, duration of diabetes, hyper-lipidemia, poor dietary habits, rural lifestyle, poor medication adherence and low education are the triggering factors of poor glycaemic control [12,13,14,15,16]. Similarly, poor self-care, depression, cognitive impairment, activities of daily living and pain are also correlated with glycaemic control [17,18,19]. In 2018, the American Diabetes Association highlighted a dire need for the evaluation of multiple geriatric domains including the medical, functional, psychological and societal domain to present an outline to establish targets and management approaches for elderly diabetic patients [9]. In 2017, an estimated 6.9% (7,474,000 people) of the Pakistani population were suffering from diabetes and the country was in 10th place (expected to be in 8th place by 2045) among high burden diabetes countries worldwide [3]. Despite this rising incidence of diabetes and its associated complications, very little is known about glycaemic control among elderly T2DM patients in Pakistan. Likewise, the significant predictors of impaired glycaemic control have not been explored. Therefore, the aim of this study was to evaluate glycaemic control among elderly Pakistani T2DM patients and to identify the significant predictors of poor glycaemic control in this context.

## 2. Methods

### 2.1. Study Design and Study Setting

This descriptive, cross-sectional study was conducted at two diabetes outpatient clinics of tertiary care hospitals (i.e., Jinnah Hospital and Sir Ganga Ram Hospital) in Lahore, Punjab province, Pakistan. Jinnah Hospital is the second largest teaching hospital in the city with 1100 beds. The hospital has a well-established outpatient diabetes clinic named the Jinnah-Allama Iqbal Institute of Diabetes and Endocrinology (JAIDE). The second hospital is the Sir Ganga Ram Hospital which was established in 1921 with 500 beds. The diabetic outpatient clinic of the hospital is located in the Department of Internal Medicine. Further details of the study setting are reported elsewhere [18]. 

### 2.2. Study Population and Data Collection

The sample population was elderly patients diagnosed with T2DM for 6 months or more, age ≥ 60 years, visiting the outpatient diabetes clinics and having their HbA1c test performed. Data was collected from 1 December 2015 to 28 February 2016 by convenience sampling techniques. The Raosoft sample size calculator was used to calculate the sample size [20], and the minimum sample size for this study was 385. The study was explained to patients visiting the diabetes clinics and they were asked for their written consent to participate in the study, upon agreeing. Participants were then enrolled in the study and were examined for height and weight, and their BMI was calculated. After this initial assessment, the patients were interviewed to complete the questionnaires based on the study objectives. Information about glycaemic values, socio-demographics and clinical characteristics of the patients were also taken from the patient’s charts. Exclusion criteria included those who were critically ill, suffering from accidental physical disabilities, unable to understand what is being said or asked of them, those suffering from life threatening diseases or those affected by epidemics like cholera, dengue or malaria. Patients suffering from severe dementia or Alzheimer’s or those unable to read and write in the native language were also excluded.

### 2.3. Study Variables

In this study, glycaemic control (i.e., FPG, RPG, HbA1c) was the outcome variable. Socio-demographic and clinical characteristics, presence of depression, cognitive status, physical function, frailty, nutritional status, pain and extent of self-care were independent variables. The target value for HbA1c was <7%, FPG was 80–130 mg/dL and RPG was <180 mg/dL [21]. Patients having HbA1c, FPG, and RPG levels beyond the upper threshold of the target levels were considered as having poor glycaemic control.

### 2.4. Survey Instruments

The patients were interviewed to complete the questionnaire that comprised of ten parts. The first and second part contained information about the socio-demographic and clinical characteristics of the patients, respectively. The third and fourth parts were the instruments for the measurement of physical function (PF), namely the Barthel Index (BI) and the Lawton Instrumental Activities of Daily Living (IADL) Scale. The fifth and sixth parts were comprised of the scales used to measure pain and frailty, namely the Clinical Frailty Scale (CFS) and the Iowa Pain Thermometer Scale (IPT). The seventh and eighth parts determined whether there was clinically significant depression and/or cognitive impairment through the Geriatric Depression Scale (GDS) and the Montreal Cognitive Assessment (MoCA) tool. The last two parts aimed to measure malnutrition and self-care via the Mini Nutritional Assessment Scale—Short Form (MNA—SF) and the Self Care Inventory—Revised Version (SCI-R). Scoring assumptions of the scales in the survey are described in Supplementary Table S1. 

The questionnaires were translated into Urdu (the national language of Pakistan) by the standard method of forward–backward translation [22]. The translated versions were pilot tested by administering to 10% of the target population. The internal consistency of the study tools was tested through Cronbach alpha (α) and is presented in Table 1.

### 2.5. Statistical Analysis

The Statistical Package for Social Sciences program (SPSS) version 21 (IBM, SPSS Statistics for Windows, version 21.0. Armonk, NY, USA: IBM Corp.) was used for data entry and analyses. For categorical variables, descriptive statistics were carried out by providing the counts (*n*) and proportions (%), whereas the continuous variables were described as means and standard deviations (SD). The normality of continuous data was assessed using the Kolmogorov–Smirnov test. To assess the association between the categorical dependent variable (impaired glycaemic control; yes = 1, no = 0) and the independent variables, simple logistic regression analysis was used [23]. Multiple logistic regression analysis was then carried out to segregate the true predictors or independent factors for statistically significant variables of univariate analysis [23]. For each predictor variable, the adjusted odd ratios (AOR), 95% confidence interval (CI), beta, standard error, and *p*-value were reported. A *p*-value of <0.05 was considered to be statistically significant.

### 2.6. Ethical Approval and Consent to Participate

The Code of Ethics of the Declaration of Helsinki was applied to the design and conduct of our study. Ethical approval was gained from the Pharmacy Research Ethics Committee (PREC) at the Islamia University of Bahawalpur (Reference: 11–2015/PREC, dated 20 October 2015). The purpose of the study was explained to each respondent before conducting the interview. Participants were required to read the “respondent information pack” and were given the opportunity to ask questions about the study before written informed consent was obtained and the administration of the questionnaire began.

## 3. Results

### 3.1. Patient Characteristics and Glycaemic Control

A total of 490 elderly T2DM patients were approached. Out of these, 400 patients agreed to participate, yielding a response rate of 81.6% with 46.3% (*n* = 185) men and 53.8% (*n* = 215) women. The mean age was 64 ± 5.5 years and the mean duration of education was 7.4 ± 4 years. More than half of the participants (52.8%, *n* = 221) were 60–62 years old, 21.3% (*n* = 85) were 63–65 years old and 26% (*n* = 104) were more than 65 years old. Nearly three-quarters (71.5%, *n* = 286) of patients were married. Over half of the participants (55%, *n* = 220) were unemployed.

In terms of glycaemic control, under half of males (40%, *n* = 74) and one-quarter of females (25.6%, *n* = 55) met the target HbA1c levels. The mean HbA1c level amongst all participants was 7.8 ± 1.5%. However, nearly two-thirds (64.2%, *n* = 145) of patients aged ≥62 years had impaired HbA1c levels. HbA1c levels were also higher in 229 (67.4%) patients with comorbidities and 227 (73.5%) patients with diabetes complications. A large proportion of patients with severe depression (90.6%, *n* = 77), mild cognitive impairment (MCI) (79.6%, *n* = 214) and low PF (72.5%, *n* = 206) failed to meet target HbA1c levels. Likewise, over three-quarters of patients with malnutrition (78.9%, *n* = 71) and severe pain (78.9%, *n* = 71) had HbA1c above target levels. 

Table 2 provides the socio-demographic and clinical characteristics of patients along with their corresponding glycaemic values.

Note: Several papers have been generated from this large study. For details on the socio-demographic and clinical characteristics of patients, please refer to our article published elsewhere [18]. It is important to note that the focus of this paper is significantly different from that previously reported in terms of objectives and outcomes.

### 3.2. Summary of Glycaemic Control

Close to one-third (32.2%, *n* = 129) of patients had HbA1c levels at target, whereas FPG and RPG levels were within the target range among 147 (36.8%) and 108 (27%) of patients, respectively (Table 3).

### 3.3. Predictors of Impaired HbA1c (Glycaemic Control)

After adjusting the predictors of impaired glycaemic control (HbA1c) in the univariate analysis (please refer to Supplementary Table S2), the factors which remained significant in the multiple logistic regression analysis were: poor diabetes self-care (AOR 0.96; 95% CI 0.95, 0.98), not prescribed with oral hypoglycaemic agents (OHA) (AOR 6.22; 95% CI 2.09, 18.46), facing frequent attacks of hypoglycaemia (AOR 2.53; 95% CI 1.34, 4.81) and falling tendency (AOR 0.19; 95% CI 0.10, 0.36) (Table 4).

## 4. Discussion

In this study, most of the study participants (three-quarters) were up to 65 years of age while more than half of the participants (52.8%) were between 60–62 years of age. In Pakistan, the life expectancy is about 66.4 years, and this is coherent with our findings [24]. Glycaemic control among most of the participants was reported to be suboptimal. The high incidence of poor glycaemic control among these Pakistani T2DM patients is highly concerning and indicates that the elderly in Pakistan are at increased risk of diabetes-associated complications and poor quality of life. Our findings are in line with the findings of previous studies from other emerging nations including Saudi Arabia [15], Uganda [14], Ethiopia [13] and Palestine [16]. In most of the studies, old age, duration of diabetes, poor medication adherence, hyper-lipidemia and absence of standard dietary plans were the most common reasons of poor glycaemic control [12,13,14,15,16]. 

In this study, patients with a low level of education had suboptimal control of their diabetes, which was reflected in high blood glucose levels. The likely explanation is that Pakistani patients with low education also have low health-literacy rates and are likely unaware of blood glucose monitoring techniques. They are likely to have a lower level of knowledge of the importance of achieving targets for blood glucose control and the pharmaco-therapeutic management of diabetes [25]. Our study supports previous work demonstrating the impact and value in educating patients on diabetes management [13,25]. Our study also reports that patients with comorbidities (for example cardiovascular disorders, hepatitis C, urinary disorders etc.) and diabetes complications (for example nephropathy, neuropathy etc.) had poor blood glucose management. The presence of these additional anomalies may result in worsening of the diabetes and reductions in physical strength and self-managing ability. This ultimately results in poor glycaemic control [26].

Our findings also demonstrate that patients with severe depression, MCI, low PF, malnutrition and impaired activities of daily living had poor glycaemic control. Compromised self-care, patients not prescribed medicines (OHA), frequent attacks of hypoglycaemia and falling tendency were found to be the statistically significant predictors of impaired HbA1c control among study participants. The study patients with depression and MCI are likely to have compromised mental states and diminished motivation which renders them unable to remember their medicines and testing and general diabetes-related self-management in order to maintain optimal glycaemic control [17,27]. Similar findings have been illustrated in other studies where significant association has been observed between depression, cognitive decline and glycaemic control [18,28,29]. Malnutrition may lead to poor physical functioning and reduced activities of daily living. Consequently, patients are unable to carryout self-management activities, leading to the poor glycaemic control demonstrated in this study [30,31].

Diabetes self-care is a fundamental requirement for optimal diabetes management. In our study, compromised self-care in elderly Pakistani diabetic patients was a determinant of poor HbA1c control. A likely reason for this is that a patient who has poor diabetic self-care is likely to demonstrate negligence in performing regular glucose checks, sticking to a pre-defined diet plan and taking their medicines properly, resulting in an uncontrolled glycaemic state [17,27]. Similarly, not being prescribed OHA therapy was also a predictor of poor HbA1c control in our study. This makes absolute sense and there are many potential reasons for this finding, although the authors are only able to speculate. One reason could be the “fear factor” of insulin administration through injection among the patients prescribed with insulin apart from OHA. This may result in poor adherence to therapy, leading to hyperglycaemia [32], while on the other hand, those injecting insulin could suffer from nocturnal hypoglycaemia [33]. In any case, the consequences are very likely to be detrimental to the long-term health of patients. A narrative review showed patients that were prescribed OHAs were more adherent to the therapy than those that were prescribed insulin and had greater control of their HbA1c levels [34]. Hypoglycaemic attacks and frequent falls have also been reported as predictors of poor HbA1c control in our study. The literature suggests both factors are interconnected so that attacks of hypoglycaemia may led to diminished energy levels and aggravation of physical disabilities, thereby increasing the tendency of diabetics having falls [35]. The hypoglycaemic events and resultant falls may make patients fearful and to prevent further attacks, patients are likely to take in excessive calories, leading to hyperglycaemia [36]. A similar finding was reported in a review in which people with diabetes tend to eat more to prevent attacks of hypoglycaemic [37].

There are limitations of this study. The findings are restricted to older adults with diabetes and the findings cannot be generalized to the younger population. Similarly, the sample was drawn from two public sector hospitals. The characteristics of patients attending private clinics could be different from the study population. The cross-sectional study design where a thorough understanding the impact of impaired glycaemic control among elderly patients requires studies that are longitudinal in nature. Having said this, HbA1c provides a sense of the stability of glycaemic control for the previous 12 weeks. However, it is suggested that future researchers should perform longitudinal studies exploring the long term impact of sub-optimal glycaemic control in elderly diabetic patients. 

## 5. Conclusions

Poor glycaemic control prevailed among the majority of elderly Pakistani diabetic patients in our study. Compromised self-care, frequent attacks of hypoglycaemia, patients not prescribed oral hypoglycaemic agents and falling tendency were found to be the independent predictors of poor glycaemic control in this cohort. 

## 6. Implications for Policy and Practice

Predictors of poor glycaemic control should be considered by healthcare professionals when resolving hurdles to diabetes management through targeting multifaceted interventions to assist with the reduction in HbA1c levels.

For older adults, diabetic self-care training is essential and must involve home-based glucose and blood pressure monitoring, education about possible diabetic related impairment and its management.

Trained and educated family members and caregivers could further facilitate elderly patients to achieve better glycaemic control at the same time as improving their functional and clinical status. Family members and caregivers can not only provide social and emotional support to the patients but can also help them in monitoring blood glucose level, injecting insulin, driving them to medical appointments, initiating and maintaining dietary and exercise schedules and assisting with the decision to follow medical recommendations.

## Figures and Tables

**Table 1:** Internal consistency and reliability of the translated versions of the data collection tools used in the pilot study.

Data Collection Tool	Internal Consistency (α)
BI	0.79
LIAD	0.88
GDS-15	0.89
SCI-R	0.76
MNA-SF	0.65

BI = Barthel Index, LIAD = Lawton Instrumental Activities of Daily Living Scale, GDS-15 Geriatric Depression Scale, SCI-R = Self Care Inventory—Revised Version, MNA-SF = Mini Nutritional Assessment—Short Form.

**Table 2:** Patient characteristics and glycaemic control.

Study Variables	HbA1c at Target Level		FPG within Target Range		RPG within Target Range	
	Yes Patients *n* (%)	No Patients *n* (%)	Yes Patients *n* (%)	No Patients *n* (%)	Yes Patients *n* (%)	No Patients *n* (%)
**Gender**						
Male	74 (40)	111 (60)	81 (43.8)	104 (56.2)	56 (30.3)	129 (69.7)
Female	55 (25.6)	160 (74.4)	66 (30.7)	149 (69.3)	52 (24.2)	163 (75.8)
**Age ≥ 62 years**						
Yes	81 (35.8)	145 (64.2)	89 (39.4)	137 (60.6)	63 (27.9)	163 (72.1)
No	48 (27.6)	126 (72.4)	58 (33.3)	116 (66.7)	45 (25.9)	129 (74.1)
**Education**						
Primary	46 (25.5)	142 (75.5)	57 (30.3)	131 (69.7)	41 (21.8)	147 (78.2)
Secondary	29 (39.7)	44 (60.3)	37 (50.7)	36 (49.3)	24 (32.9)	49 (67.1)
Tertiary	35 (47.9)	38 (52.1)	32 (43.8)	41 (56.2)	25 (34.2)	48 (65.8)
Illiterate	19 (28.8)	47 (71.2)	21 (31.8)	45 (68.2)	18 (27.3)	48 (72.7)
**Marital Status**						
Married	93 (32.5)	193 (67.5)	112 (39.2)	174 (60.8)	80 (28)	206 (72)
Unmarried	1 (16.7)	5 (83.3)	1 (16.7)	5 (83.3)	1 (16.7)	5 (83.3)
Divorced	2 (32.3)	4 (66.7)	3 (50)	3 (50)	1 (16.7)	5 (83.3)
Widowed	33 (32.4)	69 (67.6)	31 (30.4)	71 (69.6)	26 (25.5)	76 (74.5)
**Employment**						
Employed	11 (23.4)	36 (76.6)	13 (27.7)	34 (72.3)	10 (21.3)	37 (78.7)
Self-employed	26 (48.1)	28 (51.9)	29 (53.7)	25 (46.3)	21 (38.9)	33 (61.1)
Unemployed	61 (27.7)	159 (72.3)	67 (30.5)	153 (69.5)	53 (24.1)	167 (75.9)
Pensioner	31 (39.2)	48 (60.8)	38 (48.1)	41 (51.9)	24 (30.4)	55 (69.6)
**Living**						
With family	124 (32.5)	257 (67.5)	143 (37.5)	238 (62.5)	105 (27.6)	276 (72.4)
Solitary	5 (26.3)	14 (73.7)	4 (21.1)	15 (78.9)	3 (15.8)	16 (84.2)
**Comorbidities**						
Yes	111 (32.6)	229 (67.4)	125 (36.8)	215 (63.2)	95 (27.9)	245 (72.1)
No	18 (30)	42 (70)	22 (36.7)	38 (63.3)	13 (21.7)	47 (78.3)
**Comorbidity Type**						
CVD disorders	86 (33)	175 (67)	95 (36.4)	166 (63.6)	72 (27.6)	189 (72.4)
Hepatic disorders (Hepatitis C)	4 (67.8)	2 (33.3)	3 (50)	3 (50)	1 (16.7)	5 (83.3)
CVD/urinary disorders	8 (40)	12 (60)	10 (50)	10 (50)	10 (50)	10 (50)
**Number of Comorbidities**						
None	18 (30)	42 (70)	22 (36.7)	38 (63.3)	13 (21.7)	47 (78.3)
One	95 (33.6)	188 (66.4)	103 (36.4)	180 (63.6)	80 (28.3)	203 (71.7)
Two	14 (28)	36 (72)	19 (38)	31 (62)	13 (26)	37 (74)
Three	2 (28.6)	5 (71.4)	3 (42.9)	4 (57.1)	2 (28.6)	5 (71.4)
**Diabetes Complications**						
Yes	82 (26.5)	227 (73.5)	88 (28.5)	221 (71.5)	66 (21.4)	243 (78.6)
No	47 (51.6)	44 (48.4)	59 (64.8)	32 (35.2)	42 (46.2)	49 (53.8)
**Diabetes Complication Type**						
Peripheral neuropathy	64 (26.8)	175 (73.2)	72 (30.1)	167 (69.9)	54 (22.6)	185 (77.4)
Retinopathy/peripheral neuropathy	11 (26.2)	31 (73.8)	8 (19)	34 (81)	7 (16.7)	35 (83.3)
Peripheral neuropathy/nephropathy	3 (100)	0 (0)	3 (100)	0 (0)	3 (100)	0 (0)
**Number of Diabetes Complications**						
None	47 (51.6)	44 (48.4)	59 (64.8)	32 (35.2)	42 (46.2)	49 (53.8)
One	67 (26.5)	186 (73.5)	74 (29.2)	179 (70.8)	56 (22.1)	197 (77.9)
Two	14 (29.3)	34 (70.8)	12 (25)	36 (75)	10 (20.8)	38 (79.2)
Three	1 (12.5)	7 (87.5)	2 (25)	6 (75)	0 (0)	8 (100)
**BMI**						
Underweight	3 (25)	9 (75)	3 (25)	9 (75)	3 (25)	9 (75)
Normal	85 (31.4)	186 (68.6)	96 (35.4)	175 (64.6)	71 (26.2)	200 (73.8)
Overweight	39 (35.1)	72 (64.9)	45 (40.5)	66 (59.5)	32 (28.8)	79 (71.2)
Obese	2 (33.3)	4 (66.7)	3 (50)	3 (50)	2 (33.3)	4 (66.7)
**On Insulin**						
Yes	44 (25.7)	127 (74.3)	47 (27.5)	124 (72.5)	32 (18.7)	139 (81.3)
No	85 (37.1)	144 (62.9)	100 (43.7)	129 (56.3)	76 (33.2)	153 (66.8)
**On OHA**						
Yes	78 (43.3)	102 (56.7)	91 (50.6)	89 (49.4)	71 (39.4)	109 (60.6)
No	51 (23.2)	169 (76.8)	56 (25.5)	164 (74.5)	37 (16.8)	183 (82.2)
**On Combination of Insulin and OHA**						
Yes	7 (14.3)	42 (85.7)	9 (18.4)	40 (81.6)	5 (10.2)	44 (89.8)
No	122 (34.8)	229 (65.2)	138 (39.3)	213 (60.7)	103 (29.3)	248 (70.7)
**Attacks of Hypoglycaemia**						
Yes	103 (40.4)	152 (59.6)	107 (42)	148 (58)	88 (34.5)	167 (65.5)
No	26 (17.9)	119 (82.1)	40 (27.6)	105 (72.4)	20 (13.8)	125 (86.2)
**Falling Tendency**						
Yes	69 (55.2)	56 (44.8)	66 (52.8)	59 (47.2)	57 (45.6)	68 (54.4)
No	60 (21.8)	215 (78.2)	81 (29.5)	194 (70.5)	51 (18.5)	224 (81.5)
**Other Variables**						
**Depression ^a^**						
No depression	75 (57.7)	55 (42.3)	87 (66.9)	43 (33.1)	62 (47.7)	68 (52.3)
Mild depression	25 (30.5)	57 (69.5)	31 (37.8)	51 (62.2)	22 (26.8)	60 (73.2)
Moderate depression	21 (20.4)	82 (79.6)	20 (19.4)	83 (80.6)	15 (14.6)	88 (85.4)
Severe depression	8 (9.4)	77 (90.6)	9 (10.6)	76 (89.4)	9 (10.6)	76 (89.4)
**Cognitive Status ^b^**						
Normal cognitive function	74 (56.5)	57 (43.5)	95 (72.5)	36 (27.5)	64 (48.9)	67 (51.1)
Mild cognitive impairment	55 (20.4)	214 (79.6)	52 (19.3)	217 (80.7)	44 (16.4)	225 (83.6)
**PF/ADL ^c,d^**						
Physical/functional independence	21 (52.5)	19 (47.5)	23 (57.5)	17 (42.5)	18 (45)	22 (55)
Slight dependence	62 (45.6)	74 (54.4)	69 (50.7)	67 (49.3)	48 (35.3)	88 (64.7)
Moderate dependence	44 (21.4)	162 (78.6)	52 (25.2)	154 (74.8)	40 (19.4)	166 (80.6)
Severe dependence	2 (11.1)	16 (88.9)	3 (16.7)	15 (83.3)	2 (11.1)	16 (88.9)
Total dependence	0 (0)	0 (0)	0 (0)	0 (0)	0 (0)	0 (0)
**PF/IADL ^c,d^**						
High function/independent	65 (56)	51 (44)	61 (52.6)	55 (47.4)	41 (35.3)	75 (64.7)
Low function/dependence	78 (27.5)	206 (72.5)	86 (30.3)	198 (69.7)	67 (23.6)	217 (76.4)
**Frailty ^e^**						
Very fit	3 (75)	1 (25)	4 (100)	0 (0)	3 (75)	1 (25)
Well	31 (51.7)	29 (48.3)	35 (58.3)	25 (41.7)	28 (46.7)	32 (53.3)
Managing well	56 (46.3)	65 (53.7)	66 (54.5)	55 (45.5)	46 (38)	75 (62)
Vulnerable	28 (18.5)	123 (81.5)	30 (19.9)	121 (80.1)	18 (11.9)	133 (88.1)
Mildly frail	8 (18.2)	36 (81.8)	10 (22.7)	34 (77.3)	10 (22.7)	34 (77.3)
Moderately frail	3 (16.7)	15 (81.8)	2 (11.1)	16 (88.9)	3 (16.7)	15 (83.3)
Severely frail	0 (0)	2 (100)	0 (0)	2 (100)	0 (0)	2 (100)
**Nutritional Status ^f^**						
Normal nutrition	56 (50.5)	55 (49.5)	72 (64.9)	39 (35.1)	51 (45.9)	60 (54.1)
At risk of malnutrition	54 (27.1)	145 (72.9)	58 (29.1)	141 (70.9)	41 (20.6)	158 (79.4)
Malnourished	19 (21.1)	71 (78.9)	17 (19.9)	73 (81.1)	16 (17.8)	74 (82.2)
**Pain ^g^**						
No pain	11 (52.4)	10 (47.6)	15 (71.4)	6 (28.6)	8 (38.1)	13 (61.9)
Slight pain	17 (45.9)	20 (54.1)	19 (51.4)	18 (48.6)	14 (37.8)	23 (62.2)
Mild pain	28 (50.9)	27 (49.1)	30 (54.5)	25 (45.5)	21 (38.2)	34 (61.8)
Moderate pain	41 (50.6)	40 (49.4)	44 (54.3)	37 (45.7)	36 (44.4)	45 (55.6)
Severe pain	20 (20.4)	78 (79.6)	28 (28.6)	70 (71.4)	20 (20.4)	78 (79.6)
Extreme pain	6 (8.6)	64 (91.4)	7 (10)	63 (90)	4 (5.7)	66 (94.3)
Pain as bad as it could be	6 (15.8)	32 (84.2)	4 (10.5)	34 (89.5)	5 (13.2)	33 (86.8)
**Self-care Mean ^h^ (SD)**	56.1 (21.3)	38.5 (18.1)	56.6 (20.4)	36.9 (17.6)	56.6 (20.3)	39.5 (19.2)

CVD = cardiovascular disease; BMI = body mass index (underweight < 18.5 kg/m^2^, normal = 18.5−25 kg/m^2^), overweight = 25–29.9 kg/m^2^, obese ≥30 kg/m^2^); HbA1c = glycated haemoglobin; FPG = fasting plasma glucose; RPG = random plasma glucose; OHA = oral hypoglycaemic agents; PF = physical function; ADL = activities of daily living; IADL = instrumental activities of daily living; SD = standard deviation; target values: HbA1c = <7%, FPG = 80–130 mg/dL, RPG = <180 mg/dL; geriatric depression scale ^a^: <5 = normal, 5–8 = mild depression, 9–11 = moderate depression, 12–15 = severe depression; Montreal cognitive assessment ^b^: ≤26 = mild cognitive impairment, ≥26 = normal; Lawton instrumental activities of daily living scale ^c^: 0 = physical dependence, 8 = physical independence; Barthel index ^d^: 0–20 = physical dependence, 20–60 = severe dependence, 61–90 = moderate dependence, 91–99 = slight dependence, 100 = physical independence; clinical frailty scale ^e^: 1 = very fit, 2 = well, 3 = managing well, 4 = vulnerable, 5 = mildly frail, 6 = moderately frail, 7 = severely frail, 8 = very severely frail, 9 = terminally ill; mini nutritional assessment—short form scale ^f^: 12–14 = normal, 8–11 = risk of malnutrition, 0–7 = malnutrition; Iowa pain thermometer scale ^g^: 0 = no pain, 1 = mild pain, 2 = moderate pain, 3 = severe pain, 4 = extreme pain, 5 = pain as bad as it could be, 6 = most intense pain imaginable, self-care inventory—revised scale ^h^: 0 = lowest self-care, 100 = highest self-care.

**Table 3:** Glycaemic control among study participants.

	HbA1c at Target Level	FPG within Target Range	RPG within Target Range
	**Total *n* (%)**	**Total *n* (%)**	**Total *n* (%)**
Good glycaemic control	129 (32.2)	147 (36.8)	108 (27)
Poor glycaemic control	271 (67.8)	253 (63.2)	292 (73)

Target values: HbA1c = <7%, FPG = 80–130 mg/dL, RPG = <180 mg/dL.

**Table 4:** Predictors of impaired HbA1c levels: multiple logistic regression analysis.

Variables	B Value	S.E	*p*-Value	AOR (95% CI)
Self-care *	−0.035	0.010	**<0.0005**	0.96 (0.95, 0.98)
Female	−0.560	0.346	0.105	0.57 (0.29, 1.12)
Years of education *	0.014	0.042	0.742	1.01 (0.93, 1.10)
Economic dependence	−0.246	0.346	0.476	0.78 (0.39, 1.54)
Diabetes complications present	−0.075	0.546	0.891	0.93 (0.32, 2.70)
Number of diabetes complications *	0.178	0.374	0.635	1.19 (0.57, 2.49)
Not on insulin	−0.607	0.536	0.257	0.54 (0.19, 1.56)
Duration of insulin therapy *	−0.022	0.043	0.614	0.98 (0.89, 1.06)
Attacks of hypoglycaemia	0.927	0.328	**0.005**	2.53 (1.34, 4.81)
Falling tendency	−1.632	0.317	**<0.0005**	0.19 (0.10, 0.36)
Depression	0.747	0.403	0.064	2.11 (0.96, 4.65)
MCI	0.431	0.458	0.346	1.54 (0.63, 3.77)
ADL dependence	0.164	0.464	0.724	1.18 (0.47, 2.92)
Not on OHA	1.828	0.555	**0.001**	6.22 (2.09, 18.46)
IADL dependence	−0.652	0.360	0.070	0.52 (0.26, 1.05)
Frailty	0.107	0.479	0.823	1.11 (0.44, 2.84)
Pain	0.266	0.587	0.650	1.30 (0.41, 4.13)
Malnutrition	0.043	0.408	0.915	1.04 (0.47, 2.33)

S.E = standard error; AOR = adjusted odds ratio; CI = confidence interval; * = entered as continuous variable; ADL = activities of daily living; IADL = instrumental activities of daily living; OHA = oral hypoglycaemic agents; MCI = mild cognitive impairment; *p*-value < 0.05. Model summary: chi-square = 10.15, degrees of freedom = 8, *p* ˂ 0.0005, pseudo *R*^2^ = 0.439.

## Supplementary Materials

The following are available online at http://www.mdpi.com/1010-660X/55/1/21/s1, Table S1: Scoring assumptions of scales; Table S2: Predictors of impaired glycaemic control; simple logistic regression analysis.
