# Supplementary material for: Extent and Predictors of Poor Glycaemic Control among Elderly Pakistani Patients with Type 2 Diabetes Mellitus: A Multi-Centre Cross-Sectional Study
Source: Medicina (Kaunas). 2019 Jan 17;55(1):21. doi: 10.3390/medicina55010021 (PMC6358768; doi:10.3390/medicina55010021)
Supplement: Supplementary file 1 [file medicina-55-00021-s001.pdf]

Supplementary File 1; Scoring assumptions of scales

Scale name/Reference	Score	Assumptions
**Barthel Index scoring (Mahoney and Barthel, 1965)**	0-20	Physical dependence
	20-60	Severe dependence
	61-90	Moderate dependence
	91-99	Slight dependence
	100	Physical Independence
**The Lawton instrumental activities of daily living scale (Graf, 2009)**	0	Physical dependence
	8	Physical independence
**Clinical frailty scale (Research, 2004-2010)**	1	Very fit
	2	Well
	3	Managing well
	4	Vulnerable
	5	Mildly frail
	6	Moderately frail
	7	Severely frail
	8	Very severely frail
	9	Terminally ill
**Iowa pain thermometer scale and its scoring (pain, 2007)**	0	No pain
	1	Mild pain
	2	Moderate pain
	3	Severe pain
	4	Extreme pain
	5	pain as bad as it could be
	6	most intense pain imaginable.
**Geriatric depression scale (Atif et al., 2017)**	<5	Normal
	5-8	Mild depression
	9-11	Moderate depression
	12-15	Severe depression
**Mini nutritional assessment short form scale (Rubenstein et al., 2001)**	12-14	Normal
	8-11	Risk of malnutrition
	0-7	Malnutrition
**Montral cognitive assessment (Atif et al., 2017)**	≤ 26	MCI
	≥ 26	Normal
**Self-care inventory revised scale** (Khagram et al., 2013).	0	Lowest self-care
	100	Highest self-care

References

ATIF, M., SALEEM, Q. & SCAHILL, S. 2017. Depression and mild cognitive impairment (MCI) among elderly patients with type 2 diabetes mellitus in Pakistan: possible determinants. *International Journal of Diabetes in Developing Countries*, 1-9.

GRAF, C. 2009. The Lawton Instrumental Activities of Daily Living (IADL) Scale. *Medsurg Nurs,* 18, 315-6.

KHAGRAM, L., MARTIN, C. R., DAVIES, M. J. & SPEIGHT, J. 2013. Psychometric validation of the self-care inventory-revised (SCI-R) in UK adults with type 2 diabetes using data from the AT. LANTUS Follow-on study. *Health Qual Life Outcomes,* 11, 1.

MAHONEY, F. I. & BARTHEL, D. 1965. *The barthel index*. The United States patent application.

PAIN, G. 2007. *Verbal descriptor scale (pain thermometer)* [Online]. United States: Geriatric pain. Available:http://www.geriatricpain.org/Content/Assessment/Intact/Pages/PainThermometerScale.aspx (Accessed July 10 2016).

RESEARCH, D. U. F. O. M. G. M. 2004-2010. *Research/projects, clinical frailty scale* [Online]. Canada: Dalhousie University Faculty of Medicine Geriatric Medicine Research. Available: http://geriatricresearch.medicine.dal.ca/clinical_frailty_scale.htm (Accessed July 9 2016).

RUBENSTEIN, L. Z., HARKER, J. O., SALVA, A., GUIGOZ, Y. & VELLAS, B. 2001. Screening for undernutrition in geriatric practice: developing the short-form mini-nutritional assessment (MNA-SF). *J Gerontol A Biol Sci Med Sci,* 56, M366-72.

Supplementary File 2: Predictors of impaired glycemic control; Simple logistic regression analysis

In simple logistic regression analysis, the factors which were significantly associated with high HbA1c level were; female gender (OR 1.93; 95% CI 1.26, 3.96), years of education (OR 0.91; 95 % CI 0.87, 0.96), economic dependence (OR 1.58; 95% CI 1.02, 2.41), presence of diabetes complications (OR 2.96; 95% CI 1.82, 4.79), number of diabetes complications (OR 1.92; 95% CI 1.35, 2.73), not prescribed with insulin (OR 0.59; 95% CI 0.38, 0.91), not prescribed with OHA (OR 2.53; 95% CI 1.65, 3.89), not prescribed with combination therapy (OR 0.31; 95% CI 0.14, 0.72), number of diabetes complications (OR 1.92; 95% CI 1.35, 2.73), duration of insulin therapy (OR 1.06; 95% CI 1.00, 1.12), going through frequent attacks of hypoglycemia (OR 0.32; 95% CI 0.19, 0.53), falling (OR 0.23; 95% CI 0.14, 0.36), being depressed (OR 5.45; 95% CI 3.45, 8.62), having MCI (OR 5.05; 95% CI 3.20, 7.96), ADL dependence (OR 2.58; 95% CI 1.33, 4.99),

IADL dependence (OR 2.07; 95% CI 1.32, 3.25), being frail (OR 2.61; 95% CI 1.31, 5.18), being malnourished (OR 2.05; 95% CI 1.18, 3.59), feeling pain (OR 2.43; 95% CI 1.01, 5.89) and showing poor diabetes self-care (OR 0.96; 95% CI 0.95, 0.97). Table 4.4 describes the univariate OR, 95% CI and p-values, beta and standard error in detail.

Table S2. Predictors of impaired glycemic levels: Simple logistic regression analysis.

Variables	Coding	B	S.E	p-value	OR (95% CI)
**Female**					
No	0				1
Yes	1	0.662	0.217	**.002**	1.93 (1.26, 3.96)
***Age**	-	-0.019	0.020	.346	0.98 (0.94, 1.02)
***Years of education**	-	-0.092	0.026	**<.0005**	0.91 (0.87, 0.96)
**Single**					
No	0				1
Yes	1	0.043	0.238	.856	1.04 (0.65, 1.66)
**Economic dependence**					
No	0				1
Yes	1	0.459	0.215	**.033**	1.58 (1.02, 2.41)
**Living solitary**					
No	0				1
Yes	1	0.301	0.532	.572	1.35 (0.48, 3.83)
**Comorbidities present**					
No	0				1
Yes	1	-0.123	0.305	.686	0.88 (0.49, 1.61)
***Number of comorbidities**	-	0.043	0.183	.814	1.04 (0.73, 1.49)
**Diabetes complications present**					
No	0				1
Yes	1	1.084	0.246	**<.0005**	2.96 (1.82, 4.79)
***Number of diabetes complications**	-	0.651	0.180	**<.0005**	1.92 (1.35, 2.73)
***Duration of diabetes**	-	0.029	0.017	.095	1.03(0.99,1.06)
**Abnormal BMI/Weight**					
No	0				1
Yes	1	-0.125	0.227	.583	0.88 (0.57, 1.38)
**Not on insulin**					
No	0				1
Yes	1	-0.533	0.222	**.016**	0.59 (0.38, 0.91)
**Not on OHA**					
No	0				1
Yes	1	0.930	0.219	**<.0005**	2.53 (1.65, 3.89)
**Not on combination of insulin and OHA**					
No	0				1
Yes	1	-1.162	0.423	**.006**	0.31 (0.14, 0.72)
***Duration of insulin therapy**	-	0.056	0.028	**.043**	1.06 (1.00, 1.12)

**Attacks of hypoglycemia**					
No	0				1
Yes	1	-1.132	0.251	**<.0005**	0.32 (0.19, 0.53)
**Falling**					
No	0				1
Yes	1	-1.485	0.232	**<.0005**	0.23 (0.14, 0.36)
**Depression**					
No	0				1
Yes	1	1.696	0.234	**<.0005**	5.45 (3.45, 8.62)
**MCI**					
No	0				1
Yes	1	1.620	0.232	**<.0005**	5.05 (3.20, 7.96)
**ADL dependence**					
No	0				1
Yes	1	0.947	0.337	**.005**	2.58 (1.33, 4.99)
**IADL dependence**					
No	0				1
Yes	1	0.729	0.229	**.001**	2.07 (1.32, 3.25)
**Frailty**					
No	0				1
Yes	1	0.959	0.350	**.006**	2.61 (1.31, 5.18)
**Malnutrition**					
No	0				1
Yes	1	0.720	0.284	**.011**	2.05 (1.18, 3.59)
**Pain**					
No	0				1
Yes	1	0.889	0.451	**.049**	2.43 (1.01, 5.89)
***Self-care**	-	-0.045	0.006	**<.0005**	0.96 (0.95,0 .97)

BMI= Body mass index, HbA1c= Glycated hemoglobin, OHA= Oral hypoglycemic agents, PF= Physical function, ADL= Activities of daily living, IADL= Instrumental activities of daily living, * continuous variable.
